# Can analysis of performance and neuromuscular recoveries from repeated sprints shed more light on its fatigue-causing mechanisms?

**DOI:** 10.3389/fphys.2015.00005

**Published:** 2015-01-28

**Authors:** Olivier Girard, Franck Brocherie, Grégoire P. Millet

**Affiliations:** Department of Physiology, Faculty of Biology and Medicine, Institute of Sport Sciences, University of LausanneLausanne, Switzerland

**Keywords:** repeated-sprint ability, recovery, neuromuscular fatigue, neural drive, sprint-matching

In team sports, game decisive events are often reliant on transient repeated-sprint ability (RSA), which refers to the ability to produce the best possible average sprint performance over a series of sprints (<10 s), separated by short (<60 s) recovery periods (Bishop et al., 2011). Researches on RSA, particularly focusing on factors contributing to fatigue (Girard et al., 2011) and interventions (e.g., training, ergogenic aids, altitude) likely to improve this fitness component (Bishop et al., 2011; Billaut et al., 2013), are undergoing unprecedented popularity. Although differences exist in terms of sprint duration (4–10 s) or distance (10–40 m) and recovery time (10–30 s) or nature (passive or active) between RSA protocols, a single set of 5–15 maximal “all-out” efforts (i.e., close-loop design) is generally used to assess performance or fatigue resistance. Compared to time trials (i.e., possibility to constantly adjust mechanical performance) or time to exhaustion tasks (i.e., option of voluntarily ending exercise; open-loop design), one advantage of the RSA test model is to circumvent the confounding effects associated with pacing.

With the repetition of maximal efforts, muscle fatigue develops (i.e., reversible decline in muscle force production), arising from a complex interaction between muscular perturbations and neural adjustments so that no singular isolated factor likely represents a direct causative mechanism explaining the rate of decline in peak sprint speed (running) or peak/mean power output (cycling) during RSA protocols (Girard et al., 2011). In addition to large perturbations in peripheral physiological state with repeated sprinting, when substantial fatigue levels are incurred (i.e., sprint decrement score >10%), reductions in mechanical performance and in the amplitude of quadriceps EMG signals [Root Mean Square (RMS) activity] often coincide, implying that motor unit activity (i.e., a decrease in recruitment; firing rate; or both) may also become suboptimal (Mendez-Villanueva et al., 2008; Girard et al., 2011; Brocherie et al., 2014). Very recently, RSA investigations have been conducted under elevated environmental stress (heat or hypoxia) or where the degree of fatigue at exercise start was manipulated to more thoroughly understand the nature of the underlying mechanisms. The consistent finding was that acute moderate hypoxia (i.e., a fraction of inspired oxygen of 13.8%; Billaut et al., 2013) or the induction of pre-existing locomotor muscle fatigue (i.e., following a 10-min neuromuscular electrical stimulation protocol of the quadriceps; Hureau et al., 2014) caused significant parallel reductions in RMS activity of the active musculature and in power output with cycle-sprint repetitions (i.e., their magnitudes exceeded those of control situations), while the amount of peripheral quadriceps fatigue incurred at exercise termination was similar. The interpretation was that feedback from fatiguing muscles plays an important role in the determination of central motor drive and force output, so that the development of peripheral muscle fatigue is confined to a certain level (also referred as a “critical” threshold) so as not to surpass a sensory tolerance limit.

Because the modifications in muscle recruitment patterns are highly influenced by changes in RSA performance, it can be argued that muscle “de-recruitment” with sprint repetitions may not be the cause but rather the consequence of progressive decreases in velocity or power production. In an effort to resolve this issue, innovative approaches have emerged, either based on the determination of the power-EMG relationship during warm-up sprints that are subsequently compared to EMG changes during a RSA test (Bishop, 2012) or based on the comparison of fatigue responses during two sets of repeated sprints separated by a recovery period (i.e., few minutes) and matched for initial mechanical output (Mendez-Villanueva et al., 2008). The rationale is to determine whether a disproportionate decrease in neural drive over mechanical performance (sprint time/power output) actually occurs during RSA tests.

To delineate the neural and muscular factors driving performance recovery following repeated sprints a sprint-matching paradigm was introduced, where exercise responses during two sets of repeated cycling sprints (10 × 6-s “all out” sprints with 30 s recovery followed after 6 min of passive recovery by five 6-s sprints), matched for initial mechanical output in a “non-fatigued” (sprints 4–8) and a “fatigued” state (sprints 11–15), were actually compared (Mendez-Villanueva et al., 2007). Results indicated that there was a greater fatigability in the five repetitions of the second vs. first set, despite mechanical output produced for the initial bout of both sets (i.e., sprints 4 and 11) being similar. Furthermore, muscle activation was lower (~12%) in sprint 11 than 4, while the rate of decrease in net EMG activity was similar for the two sets of repeated sprints. Taken as a whole, this highlights that the short-term activation history of the active musculature alters the muscle recruitment pattern and fatigability during sets of repeated sprints matched for initial power output. Using the same data set, with the addition of muscle biopsies of the *vastus lateralis* obtained at rest, immediately after the 10 first sprints and after 6 min of recovery it was further demonstrated that phosphocreatine resynthesis was associated with total work done in sprint 11 (*r* = 0.79, *P* < 0.05) and total work done during sprints 11–15 (*r* = 0.67, *P* < 0.05), while EMG amplitude remained depressed (Mendez-Villanueva et al., 2013). The lower performance maintenance during subsequent repeated sprints was mostly mediated by intramuscular factors probably related to limitations in metabolic supply, as also evidenced by the disproportionate ~2-fold greater decrease in total work in relation to RMS in the second set of sprints (sprint 11–15) than in the first five sprints (sprint 1–5).

In an effort to improve our understanding of fatigue-causing mechanisms during repeated sprinting, we invite multiple-sets RSA studies to carefully evaluate the ensuing recovery rate of single- and multiple-sprint performance and return of neuromuscular markers, with special reference to restoration of central nervous system functioning and of peripheral physiological state. Furthermore, quantifying whether disproportionate decreases in neural drive or in muscle contractility occur over mechanical performance during successive sets of repeated sprints, may help to determine if the attenuation of the EMG amplitude is actually the consequence, or the cause, of slower sprint times or reduced power production. Further investigation where environmental stressing conditions could vary across successive sets of repeated sprints and/or during the between-sets intervening recovery periods may assist in clarifying this contention, accepting the premise that an increase in hypoxia severity would alter exercise-induced demands (and thereby recovery requirements) on the neuromuscular system. Under this framework, our recent comparison of the effects of an initial set of exhaustive intermittent cycling under normoxia, moderate or severe hypoxia on locomotor performance and quadriceps fatigability, and how recovery from this first exercise bout influence subsequent normoxic performance during the completion of a second set using a similar exercise mode may lead the way (Christian et al., 2012). In doing so particular attention should be paid to study perceptual recovery as well, as it may interact with feed-forward/feedback mechanisms to influence athlete preparedness for ensuing exercise bouts (Minett and Duffield, 2014).

## Conflict of interest statement

The authors declare that the research was conducted in the absence of any commercial or financial relationships that could be construed as a potential conflict of interest.
